# Chromosome-Level Assembly of the Southern Rock Bream (*Oplegnathus fasciatus*) Genome Using PacBio and Hi-C Technologies

**DOI:** 10.3389/fgene.2021.811798

**Published:** 2021-12-21

**Authors:** Yulin Bai, Jie Gong, Zhixiong Zhou, Bijun Li, Ji Zhao, Qiaozhen Ke, Xiaoqing Zou, Fei Pu, Linni Wu, Weiqiang Zheng, Tao Zhou, Peng Xu

**Affiliations:** ^1^ State Key Laboratory of Marine Environmental Science, College of Ocean and Earth Sciences, Xiamen University, Xiamen, China; ^2^ State Key Laboratory of Large Yellow Croaker Breeding, Ningde Fufa Fisheries Company Limited, Ningde, China; ^3^ Fujian Key Laboratory of Genetics and Breeding of Marine Organisms, College of Ocean and Earth Sciences, Xiamen University, Xiamen, China

**Keywords:** *Oplegnathus fasciatus*, genomic resources, PacBio, Hi-C, phylogenetic analysis

## Abstract

The Rock Bream (*Oplegnathus fasciatus*) is an economically important rocky reef fish of the Northwest Pacific Ocean. In recent years, it has been cultivated as an important edible fish in coastal areas of China. Despite its economic importance, genome-wide adaptions of domesticated *O. fasciatus* are largely unknown. Here we report a chromosome-level reference genome of female *O. fasciatus* (from the southern population in the subtropical region) using the PacBio single molecule sequencing technique (SMRT) and High-through chromosome conformation capture (Hi-C) technologies. The genome was assembled into 120 contigs with a total length of 732.95 Mb and a contig N50 length of 27.33 Mb. After chromosome-level scaffolding, 24 chromosomes with a total length of 723.22 Mb were constructed. Moreover, a total of 27,015 protein-coding genes and 5,880 ncRNAs were annotated in the reference genome. This reference genome of *O. fasciatus* will provide an important resource not only for basic ecological and population genetic studies but also for dissect artificial selection mechanisms in marine aquaculture.

## Introduction

**FIGURE 1:**
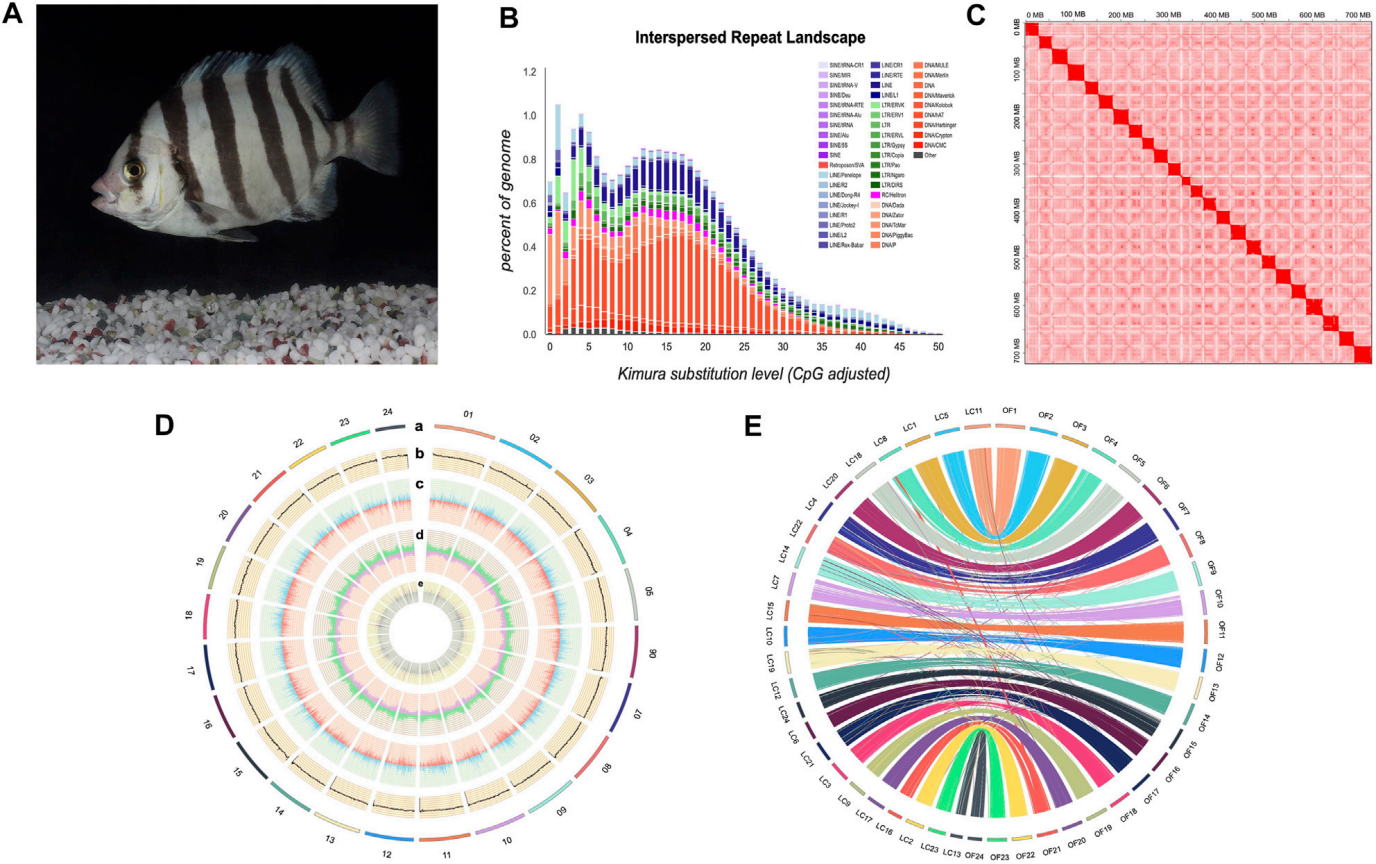
Characteristics of Oplegnathus fasciatus genome assembly. **(A)** A photograph of female *O. fasciatus* (from Zhoushan population). **(B)** Distribution of divergence rate for each type of TE in *O. fasciatus* genome. Percentages of TEs in genome (Y-axis) and Kimura divergence (X-axis). Values from 0-50 reflect older copies (right side) and new copies (left side). **(C)** Heat map of chromosomal interactions in the *O. fasciatus* genome using Hi-C data. **(D)** A circos plot of 24 chromosomes in *O. fasciatus* genome, the tracks from outside to inside are: a. Lines represent *O. fasciatus* chromosomes; b. GC content; c. Gene density; d. Repeat element density; e. ncRNA density. b-e. are drawn in nonoverlapping 100 kb sliding windows. **(E)** Circos diagram between *O. fasciatus* and *L. crocea*. Each coloured arc represents a 1 Kb fragment match between two species. We re-ordered the chromosome numbers of *L. crocea* for better illustration.

The Rock Bream (*Oplegnathus fasciatus*), inhabiting in coastal rocky reefs and feeds on invertebrates inhabiting the seabed, is an endemic marine fish in East Asia that belongs to Oplegnathidae in *Perciformes* (Figure 1A) (An et al., 2008). In recent years, *O. fasciatus* has become one of the most commercially valuable marine fishery species in China aquaculture (Xiao et al., 2019). As a sedentary species, due to habitat restrictions, *O. fasciatus* is broadly and fragmented distributed in the coastal areas of eastern China (Xiao et al., 2016a). According to the difference of genetic variation, the researchers identified the *O. fasciatus* population as northern (Jiaonan, Qingdao) and southern regions (Zhoushan, Zhejiang) of Chinese coastal waters (Xiao et al., 2016b). The different environments make them divergent in genetic structure and living habits, which provides a suitable fish model for the study of population genetic in marginal sea (Jian et al., 2017; Chen et al., 2019a). The male and female reference genome of *O. fasciatus* derived from the northern population has been reported and revealed its particularity in gender mechanism (Xiao et al., 2019; Xiao et al., 2020). However, a highly accurate reference genome of subtropical *O. fasciatus* species is still lacking, which hinders the progress of genome-scale genetic breeding and genetic studies of its temperature plasticity and adaptation at lower latitudes.

Recently, the genetic breeding of economic fish has attracted much attention, mainly aiming to improve growth rates and disease resistance under aquaculture conditions (Bangera et al., 2017; Gong et al., 2021; Zhao et al., 2021). Genome selection (GS) has become an efficient and popular breeding strategy, which depends on high-quality reference genome (Singh et al., 2018). It is worthwhile to invest time and money to obtain a reference genome more suitable for breeding populations to improve the prediction accuracy (Benevenuto et al., 2019). Our breeding work related to the growth traits of *O. fasciatus* reported recently also supports this conclusion (Gong et al., 2021). In this report, we provided a chromosome-level reference genome of *O. fasciatus* (from the southern population in the subtropical region) using a combination of the PacBio single molecule sequencing technique (SMRT) and high-through chromosome conformation capture (Hi-C) technologies. We assembled the genome sequences into 120 contigs with a total length of 732.95 Mb and a contig N50 length of 27.33 Mb. After chromosome-level scaffolding, 24 scaffolds were constructed corresponding to 24 chromosomes with a total length of 723.22 Mb (98.67% of the total length of all contigs). The availability of data is essential to support the population genetic studies, and will also provide an important resource for the upcoming breeding program of *O. fasciatus*.

## Materials and Methods

### Sample Collection, Library Construction and Sequencing

A healthy female *O. fasciatus* (from Zhoushan population) were obtained from a commercial breeding company, Ningde Fufa Aquatic Breeding (Fujian, China). All samples were collected, snap frozen in the liquid nitrogen and stored at −80°C to maintain nucleic acid integrity.

The muscle tissues were collected for DNA extraction. For the PacBio sequencing project, frozen samples were lysed in SDS digestion buffer with proteinase-K (50 µg/ml). Then, the lysates were purified using AMPure XP beads (Beckman Coulter, High Wycombe, United Kingdom) to obtain High-Molecular-Weight gDNA. Library construction and sequencing were conducted according to the manufacturer’s protocol with the PacBio RS-II platform at Novogene (Tianjin). Meanwhile, the Normal-Molecular-Weight gDNA for Illumina sequencing was extracted from the same samples using the PureLink™ Pro 96 Genomic DNA Purification Kit (Invitrogen, Shanghai, China). A pair-end library with 350 bp insert size was constructed and sequenced using the Illumina NovaSeq 6000 platform with a read length of 2 × 150 bp.

For Hi-C sequencing, The DNA was fixed by formaldehyde to maintain the conformation and the restriction enzyme (HindIII) was applied on DNA digestion, followed by repairing 5’ overhangs with biotinylated residues. After *in-situ* ligation of these fragments, DNA was reverse-cross linked and purified. Finally, sequencing of the Hi-C library was performed on an Illumina NovaSeq 6000 platform.

Additionally, 11 different tissues (heart, liver, spleen, intestine, kidney, skin, eye, gill, brain, blood, muscle) were collected to extract RNA for RNA sequencing (RNA-seq) following the protocols of the PureLink™ RNA Mini Kit (Invitrogen, Shanghai, China). The library was constructed using the Illumina standard protocol (San Diego, CA, United States) and sequenced on the Illumina HiSeq 6000 platform.

### Data Processing and Genome Assembly

Before assembly, the PacBio data was further filtered, and reads with length less than 1500 bp or low-quality were removed. For the Illumina data, adapter sequences and low-quality reads were filtered using fastp (v. 0.23.1) software. The remaining reads were used for further assembly and estimation of genome size using the K-mer analysis of the short reads (see below).

SOAPec (v. 2.01) and GenomeScope (v. 2.0) softwares were used to analyze the K-mer frequencies in the sequencing reads to obtain genome characteristics such as genome size, heterozygosity, and repeatability.

To obtain chromosome-level whole genome assembly for *O. fasciatus*, we utilized a combined approach of Illumina, PacBio and Hi-C technology for the genome assembly and chromosome-level scaffolding. Then, low-quality and duplicated reads were filtered out. The *O. fasciatus* genome was assembled using a hybrid SMRT-Illumina-HiC strategy as follows: 1) Contigs from Continuous Long-Read (CLR) clean reads were assembled using Canu (v. 2.0) (Sergey et al., 2017) with default parameters. The high-fidelity contig sets was produced by using a combination of circular consensus CLR reads, Illumina paired-end reads with sufficient overlap to merge into single extended accurate reads; 2) Purge_Dups (v. 1.2.5) was employed to resolve redundancy in the assembly; 3) The non-redundant contig sets were reordered and scaffolded using 3D-DNA pipeline (Dudchenko et al., 2017); 4) Scaffolds were fine-tuned and discordant contigs were removed from scaffolds using Juicebox (v. 1.5) (Robinson et al., 2018). Finally, we obtained a chromosome level reference genome of *O. fasciatus* containing linkage group information.

### Annotation of Genomic Repeats

Repetitive sequences of the *O. fasciatus* genome were annotated using both homology-based search and *de novo* methods. RepeatModeler (v. 2.0.1) and LTR_Finder (v. 1.07) were used to detect repeat sequences in the *O. fasciatus* genome. Combined with Repbase (v. 20,181,026; http://www.girinst.org/repbase), a repeat sequence library was constructed. RepeatMasker (v. 4.1.0) were utilized to search and classify repeats based on this library. TEclass (v. 2.1.3) was used to further annotate unclassified repeats. The built-in script buildSummary.pl from RepeatMasker (v. 4.1.0) was used to summarize Transposable Elements (TEs) annotation results. Then two scripts, calcDivergenceFromalign.pl and createRepeatLandscape.pl, were used to calculate the Kimura divergence value and draw repeated landscapes (Figure 1B). The nucleotide distances between all copies of each TE measured using the Kimura two-parameter method were compared to estimate insertion age (Schemberger et al., 2019). Tandem Repeats Finder (v. 4.09) was used to identify tandem repeats. All repetitive regions except tandem repeats were soft-masked for protein-coding gene annotation.

### Protein-Coding Gene Finding and Function Annotation

The coding sequences of genetically close species, including *L. crocea*, *L. maculatus*, *G. aculeatus*, and *P. olivaceus*, were retrieved from Ensembl and NCBI. These coding sequences were provided to the software package of Braker2 (v. 2.1.5) (Bruna et al., 2021), and then the genes in the repeat-masked reference genome were annotated with the close homologous protein model. RNA-seq data were aligned to *O. fasciatus* contigs using Blat (v. 36 × 5) and GMAP (v. 2017-11-15), and a comprehensive transcriptome database was built using PASA (v. 2.4.1) (Haas and Salzberg, 2008). The transdecoder software (v. 5.5.0) was used to predict gene structure based on ESTs evidence, and the credible gene structure annotation information was provided to train parameters for the following *de novo* gene prediction software packages: Augustus (v. 3.4.0) and GeneMark (v. 4.62). Finally, evidence from the gene finders, protein homology searches and ESTs were provided to the EvidenceModeler (v 1.1.1) (Haas and Salzberg, 2008) to obtain a comprehensive and non-redundant gene set.

For gene function annotation, we used Diamond (v. 2.0.6) to search the homologous sequences from the Swiss-Prot (http://www.uniprot.org/), TremBL (http://www.uniprot.org/) and NR protein databases. We were also subjected to GO annotation and protein family annotation by InterProScan (v. 4.8) (https://www.ebi.ac.uk/interpro/). KO terms for each gene are assigned by an online website (KAAS, https://www.genome.jp/tools/kaas/). The programs tRNAScan-SE (v. 1.3.1) and RNAmmer (v. 1.2) were used to predict tRNA and rRNA, respectively. The other ncRNAs were identified by searching against the Rfam database (http://eggnogdb.embl.de/).

Internal scripts were used to calculate GC content, gene density, repetitive element density, ncRNA density, and gene components distribution based on gff3 format file in the annotation results.

### The Completeness Assessment of Assembly and Annotation

Assembly completeness and accuracy were evaluated by multiple methods. First, the Illumina short reads were re-mapped to the genome using BWA (v. 0.7.17), and the mapping ratio was counted by samtools (v. 1.8; with the pattern “flagstat”). Then, we used the Benchmarking Universal Single Copy Orthologues (BUSCO) (v. 5.2.2) (Seppey et al., 2019) to test the integrality of the final assembly and the lineage dataset was actinopterygii_odb10. Similarly, the annotation integrity was evaluated based on the protein sequence sets using the protein pattern built into BUSCO software (v. 5.2.2).

### Phylogenetic Analysis

Single-copy genes in *O. fasciatus* and 10 related species (*G. morhua*, *L. maculatus, L. crocea, G. aculeatus*, *N. coriiceps*, *T. bimaculatus*, *O. latipes*, *C. semilaevis*, *B. petinirostris*, and *D. reio*) were identified based on gene families constructed from protein sequences of all species employing OrthoFinder (v. 2.5.4) (Emms and Kelly, 2019) software with default parameters. Single-copy ortholog proteins were aligned by MUSCLE (v. 3.8.31). Subsequently, all obtained alignments were converted to their corresponding coding DNA sequences using an in-house python script. A combined continuous ultra-long sequence was constructed from all the translated coding DNA alignments for minimum evolution (ME) phylogenetic tree construction using MEGA. The divergence time is estimated using MCMCTREE (PAML package) (Yang, 1997) based on the molecular clock data of Timetree database (Hedges et al., 2006).

## Results and Discussions

With a 100x sequencing depth sequencing reads from Illumina and Pacbio platforms, we assembled a high-quality chromosome-level reference genome from a female *O. fasciatus*. Based on the 17-kmer analysis and a dominant peak depth of 51.57, the genome size was estimated to be 751.19 Mb with the heterozygosity and repetitive sequence content were approximately 0.28 and 23.09%, respectively (Supplementary Table S1). The genome size is slightly smaller than that of the *O. fasciatus* derived from the northern population (previously reported; male ∼762 Mb and female ∼768.8 Mb) (Xiao et al., 2019; Xiao et al., 2020). A trimodal pattern was observed in the 17-mer frequency distribution analysis. The main peak (the second peak) is much higher than the other two sub peaks, indicating that there is a certain degree of heterozygosity and duplication in *O. fasciatus* genome (Supplementary Figure S1) (Manekar and Sathe, 2019).

The PacBio sequencing reads were used for *de novo* assembly of the genome. And the average read length and N50 of read length were 21,083 and 30,815 bp, respectively. The initial assembly yielded a total length of 1.1 Gb, comprising 2,789 contigs with a contig N50 length of 21.91 Mb. The genome assembly was larger than the estimated genome size of 751.19 Mb (see above), because some redundant sequences failed to be merged due to high heterozygosity (Zhang et al., 2012). After eliminating the redundancy, we obtained a final genome assembly of 732.95 Mb for the *O. fasciatus*, which is nearly equal to the estimated genome size (Table1 and Supplementary Table S1). Then, the PacBio draft assembled contigs were anchored and oriented into a chromosomal-scale assembly *via* the Hi-C scaffolding approach (Figure 1C). Finally, we generated a chromosome-level genome assembly of 732.99 Mb in length, with a contig N50 and scaffold N50 value of 27.33 and 31.10 Mb, respectively (Figure 1D and Table 1). The total length of assembly contained 24 chromosomes (the lengths ranged from 18.22 to 37.18 Mb) was 723.22 Mb, and the integration efficiency was 98.67% (Table 1 and Supplementary Table S2). The genome sizes of *O. fasciatus* (733.99 Mb) were similar with two closely related Perciformes species i.e., *L. crocea* (723.86 Mb) (Chen et al., 2019a) and *Oplegnathus punctatus* (718 Mb) (Li et al., 2021). To further verify the integrality of the assembled genome, the reads from the short-insert library were re-mapped onto the assembled genome using BWA (version 0.7.17). A total of 97.89% of the reads mapped to a reference sequence in this genome (Table 1). Additionally, we tested completeness by attempting the recovery of conserved single-copy genes from *O. fasciatus* genome by BUSCO (v. 5.0.0). Out of a database containing 3,640 single-copy protozoan orthologs, ∼97% were fully recovered from the assembly (Table 1 and Supplementary Table S3). The high integration efficiency (∼98.67%), mapping ratio (97.89%), and the recognition rate of single copy orthologs (∼97%) show that our assembly of *O. fasciatus* is of high quality, which is at the same level as some recently published high-accurate reference genomes of other marine fish (Chen et al., 2019a; Zhou et al., 2019).

**TABLE 1:** Summary of the *Oplegnathus fasciatus* genome assembly and annotation.

Genome assembly and chromosomes construction	
Contig N50 size (Mbp)	27.33
Contig number (>100 bp)	120
Contig total length (Mbp)	732.95
Scaffold N50 size (Mbp)	31.1
Scaffold number (>100 bp)	153
Scaffold total length (Mbp)	732.99
Number of chromosomes	24
Total length of chromosomes (Mbp)	723.22
Integration efficiency of Hi-C map (%)	98.67
Illumina Short reads re-mapping ratio (%)	97.89
Proportion of BUSCO in genome model (%)	97
**Gene Prediction and Annotation**	
Protein-coding gene number	27,015
Mean transcript length (bp)	3159.45
Mean exons length (bp)	167.47
Mean exons number per gene	9.27
Proportion of BUSCO in proteins model. (%)	95.6

There was a total of 224.97 Mb of consensus and nonredundant repetitive sequences obtained by a combination of known, novel and tandem repeats, occupying more than 30.69% of the whole genome assembly (Figure 1B and Supplementary Table S4). The *de novo* and homologous prediction were utilized to investigate the repeat sequences (Supplementary Table S5). TE divergence analysis suggested recent activity of DNA transposons and long terminal repeats in this genome (Figure 1B) (Wang et al., 2019). DNA transposons were the most abundant repetitive elements, spanning at least 113.54 Mb, accounting for 15.49% of the whole genome of O. fasciatus. Among them, the repetitive sequences also comprised of long interspersed elements in 44.78 Mb (LINEs; 6.11%), short interspersed nuclear elements in 1.37 Mb (SINEs; 0.19%) and long terminal repeats in 34.02 Mb (LTRs; 4.64%) (Supplementary Table S5).

**FIGURE 2:**
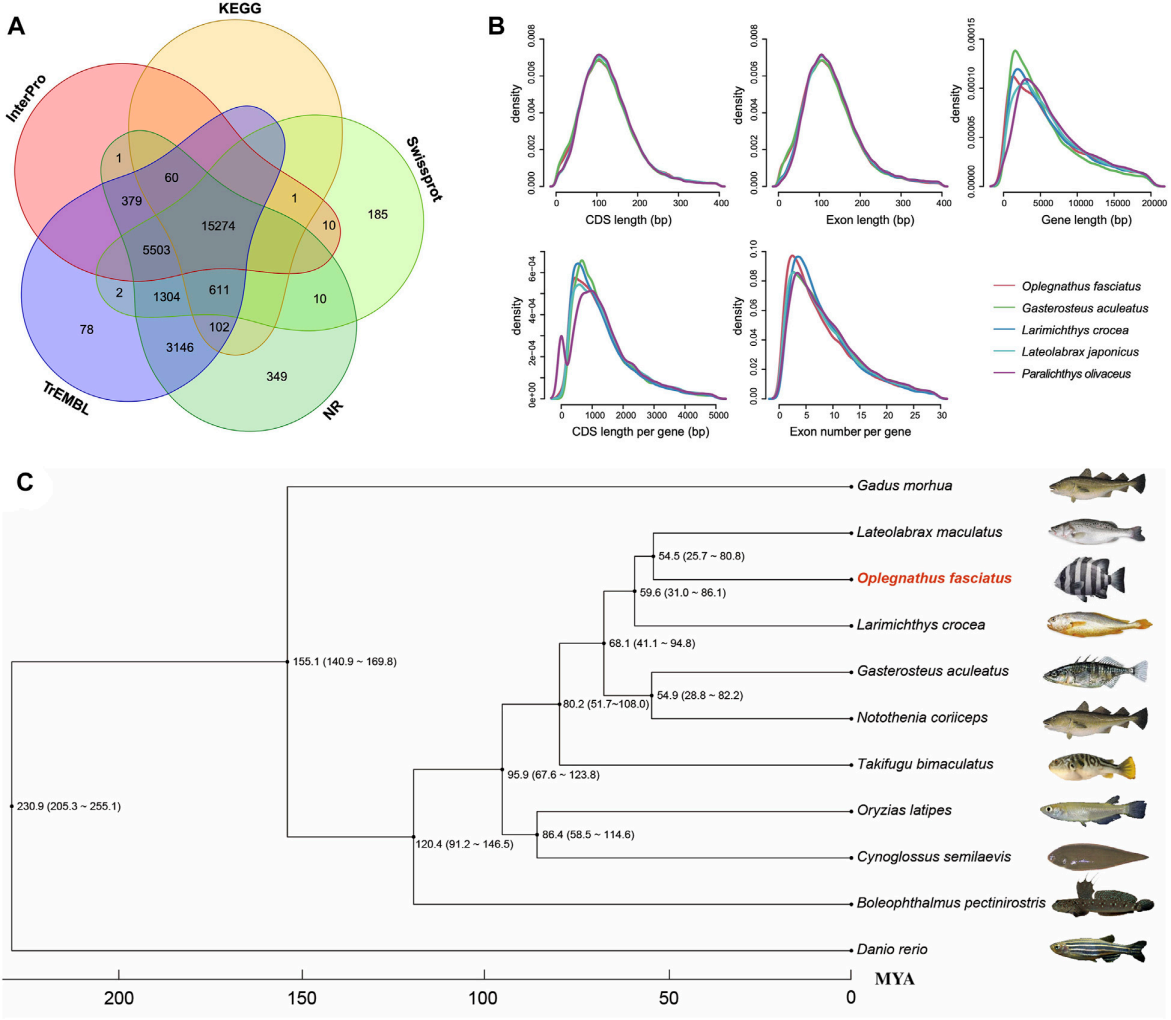
Gene annotations of the Oplegnathus fasciatus genome and phylogenetic relationships among eleven species. **(A)** Venn diagram of the functionally annotated protein-coding genes. **(B)** Gene components distribution patterns among *O. fasciatus* and related species (*G. aculeatus*, *L. crocea*, *L. maculatus*, and *P. olivaceus*). **(C)** Phylogenetic analysis among *O. fasciatus* and representative species (*G. morhua*, *L. maculatus, L. crocea, G. aculeatus*, *N. coriiceps*, *T. bimaculatus*, *O. latipes*, *C. semilaevis*, *B. petinirostris*, and *D. reio*).

A total of 27,015 nonredundant protein-coding genes were successfully yielded combining *de novo*, homologous searching and transcriptome-assisted predictions (Table 1). The statistics of the predicted gene models were compared to the homologous protein sequences of *G. aculeatus*, *L. crocea*, *L. japonicus* and *P. olivaceus*, which indicated closely distribution patterns in mRNA length, CDS length, exon length and exon number (Figure 2B and Supplementary Table S7). There were 17,054 complete ORFs, 27,015 complete transcripts and 250,306 exons detected in the *O. fasciatus* genome. The mean lengths of exon were 167.47 bp. The average exon number for each gene was 9.27 and the average length of CDS was 1551.68 bp (Supplementary Table S7). We annotated these genes against several public databases, including NR, TrEMBL, Swissprot and InterPro, resulting in 98.98, 97.94, 84.77 and 78.58% of the genes functionally assigned, respectively. Furtherly, we detected protein domains in multiple databases, and 59.76 and 59.40% of the predicted genes were annotated using GO and KEGG database, respectively. Finally, 27,015 genes were successfully functional annotated in at least one of these databases (Figure 2A and Supplementary Table S6). The number of predicted genes of *O. fasciatus* (27,015) through *de novo* prediction and homologue annotation was slightly more than others in Perciformes, such as *L. crocea* (23,657) (Chen et al., 2019a), *L. maculatus* (22,509) (Chen et al., 2019b), and previous version of *O. fasciatus* (24,003) from the northern population (Xiao et al., 2019). BUSCO analysis with protein pattern suggested that 97.2% (3,537) of the core conserved genes were detected in the *O. fasciatus* gene set, with 3,480 (95.6%) and 57 (1.6%) being identified as complete and fragmented, respectively (Table 1 and Supplementary Table S3). The results indicate that our gene structure annotation is relatively complete (Seppey et al., 2019). To verify the accuracy of the contig arrangement in 24 chromosomes, we aligned 1 K bp small fragments with 50 K bp spacing as anchors of the assembled genome against the published *L. crocea* genome to compare consistency between these two genomes. The 24 chromosomes we identified in the *O. fasciatus* genome aligned exactly against the chromosomes of the *L. crocea*, suggesting high continuity with the *O. fasciatus* genome (Figure 1E). Furthermore, four types of non-coding RNAs were identified in *O. fasciatus* genome, including 1,188 miRNA, 1,808 tRNAs, 1,793 rRNAs and 1,091 snRNAs (Table 1 and Supplementary Table S8).

To reveal the phylogenetic relationships among *O. fasciatus* and other species, a total of 2,516 single copy ortholog protein families in a 1:1:1 manner from the 10 related species (as described above) were identified and used for phylogenetic analysis (Figure 2C). Previous reports showed that *O. fasciatus* and *L. crocea* had close genetic distance and clustered in Perciformes (Eupercaria) (Xiao et al., 2019). According to our phylogenetic analysis, we further found that the divergence time between *O. fasciatus* and the common ancestor with *L. maculatus* (25.7-80.8 million years ago) was shorter than *L. crocea* (31.0–86.1 million years ago). In addition, the phylogenetic relationship between *O. fasciatus* and other fish is also consistent with previous taxonomic studies (Oh et al., 2007; Betancur-R et al., 2017), indicating that the estimation of divergence time in this study should be reasonable.

Certainly, a high-precision chromosomal genome resource is foremost to identify genetic variation underlying phenotypic traits of economic interest for aquaculture production (Chen et al., 2019a; Chen et al., 2019b; Zhou et al., 2019). The sex determination mechanism of *O. fasciatus* has attracted wide attention of researchers in recent years (Xiao et al., 2020). Previous cytogenetic analysis of *O. fasciatus* has shown that it has morphologically distinguishable sex chromosomes, and there are differences in the number of male (2n = 47; X1X2Y) and female (2n = 48; X1X1X2X2) chromosomes (Xu et al., 2019). Xiao et al. have reported the sex-related comparative genomics study of the northern population of *O. fasciatus* (Xiao et al., 2020), but it is still unclear whether the key genetic locus is restricted by geography. Our report also provides an important resource for further research on the mechanism of gender determination.

## Conclusion

Here, we report a highly accurate chromosome-level genome assembly of *O. fasciatus* from the southern population in the subtropical region based on PacBio and Hi-C technologies. The genome size (732.99 Mb) is slightly smaller than that of the *O. fasciatu*s derived from the northern population. A total of 27,015 gene structures were annotated using the strategy of multi-evidence combination. We found that the divergence time between *O. fasciatus* and the common ancestor with *L. maculatus* was shorter than *L. crocea*. The genome data created in this study will serve as valuable resources for species diversity and population genetic research, and will further promote the progress of genome-scale genetic breeding and genetic studies of its temperature plasticity and adaptation at lower latitudes.

## Code Availability

### Genome Survey and Assembly

1) SOAPec: version 2.01; -k 17 -l 11; 2) GenomeScope: version 2.0; all parameters were set as default; 3) Canu: version 2.0; all parameters were set as default; 4) Racon: version 1.4.3; all parameters were set as default; 5) NextPolish: version 1.4.0; job_type = local; task = 1212; rewrite = no; rerun = 3; sgs_options = -max_depth 100 -bwa; 6) Purge_Dups: version 1.2.5; all parameters were set as default.

### Genome Annotation:

1) RepeatMasker: version 4.1.0; -no_is -nolow -norna -gff -poly -html; 2) RepeatModeler: version 2.0.1; -database genome -engine ncbi; 3) TEclass: version 2.1.3; all parameters were set as default; 4) TRF: version 4.09; 2 7 7 80 10 50, 500 -m -f -d; 5) Braker2: version 2.1.5; -gff3 –species --genome --prot_seq --bam --prg = *g*th --softmasking 6) Transdecoder: version 5.5.0; -t transcripts.fasta; 7) PASA: version 2.4.1; -c alignAssembly.config -C -R -g genome -t transcripts.fasta.clean -T -u transcripts.fasta --ALIGNERS blat, gmap; 8) Diamond: version 2.0.6; --outfmt 5; 9) EVidenceModeler: version 1.1.1; --gene_predictions --protein_alignments --transcript_alignments --segmentSize 100000 --overlapSize 10000 –weights weights.txt.

## Data Availability

The datasets presented in this study can be found in online repositories. The names of the repository/repositories and accession number(s) can be found below: https://www.ncbi.nlm.nih.gov/, PRJNA778612, https://figshare.com/, https://doi.org/10.6084/m9.figshare.16950832.

## Abbreviations

*B. petinirostris:* Blue Spotted Mudskipper, *Boleophthalmus petinirostris*; *C. semilaevis:* Half-Smooth Tongue Sole, *Cynoglossus semilaevis*; BUSCO, Benchmarking Universal Single Copy Orthologues; CLR, Continuous Long-Read; *D. reio:* Zebrafish, *Danio reio*; GS, Genome selection; *G. aculeatus:* Three-Spined Stickleback, *Gasterosteus aculeatus*; *G. morhu:* Atlantic Cod, *Gadus morhua*; Hi-C, High-through chromosome conformation capture; *L. crocea*: Large Yellow Croaker, *Larimichthys crocea*; *L. japonicus:* Chinese Seabass, *Lateolabrax maculatus*; PacBio SMRT, ME, Minimum Evolution; *N. coriiceps*: Antarctic rock cod, *Notothenia coriiceps*; PacBio Single molecule sequencing technique; *O. fasciatus:* Rock Bream, *O. latipes:* Japanese Rice Fish, *Oryzias latipes*; *Oplegnathus fasciatus*; *P. olivaceus:* Olive Flounder, *Paralichthys olivaceus*; *T. bimaculatus:* Pufferfish, *Takifugu bimaculatus*; TEs, Transposable Elements.
