# Supplementary material for: Chromosome-Level Assembly of the Southern Rock Bream (Oplegnathus fasciatus) Genome Using PacBio and Hi-C Technologies
Source: Front Genet. 2021 Dec 21;12:811798. doi: 10.3389/fgene.2021.811798 (PMC8724560; doi:10.3389/fgene.2021.811798)
Supplement: Supplementary file 1 [file Table1.DOCX]

| **Table S1.** The data statistic of 17-mer analysis and heterozygosity of *O. fasciatus* genome. | | | | | | | | |  |
| --- | --- | --- | --- | --- | --- | --- | --- | --- | --- |
| Sample | Kmer | kmer depth | kmer number | Genome size (M) | | Revised Genome size (M) | Heterozygosity (%) | Repeat rate (%) | |
| muscle | 17 | 51.57 | 40,626,5-40,528 | | 757.00 | 751.19 | 0.28 | 23.09 | |
