# Supplementary material for: Chromosome-Level Assembly of the Southern Rock Bream (Oplegnathus fasciatus) Genome Using PacBio and Hi-C Technologies
Source: Front Genet. 2021 Dec 21;12:811798. doi: 10.3389/fgene.2021.811798 (PMC8724560; doi:10.3389/fgene.2021.811798)
Supplement: Supplementary file 2 [file Table8.DOCX]

| **Table S8.** Summary statistics of non-coding RNA. | | | | | |
| --- | --- | --- | --- | --- | --- |
| **Type** |  | **Copy** | **Average length (bp)** | **Total length (bp)** | **% of genome** |
| **miRNA** |  | 1,188 | 103.40 | 122,835 | 0.01598 |
| **tRNA** |  | 1,808 | 75.38 | 136,281 | 0.01773 |
| **rRNA** | **rRNA** | 1,793 | 168.31 | 301,787 | 0.03926 |
|  | **18S** | 35 | 1209.89 | 42,346 | 0.00551 |
|  | **28S** | 163 | 458.40 | 74,719 | 0.00972 |
|  | **5.8S** | 21 | 155.24 | 3260 | 0.00042 |
|  | **5S** | 1,574 | 115.29 | 181,462 | 0.02361 |
| **snRNA** | **snRNA** | 1,091 | 146.65 | 161,316 | 0.02099 |
|  | **CD-box** | 152 | 130.47 | 19,831 | 0.00258 |
|  | **HACA-box** | 78 | 149.24 | 11,641 | 0.00151 |
|  | **splicing** | 861 | 148.28 | 127,669 | 0.01661 |
