# Supplementary material for: Chromosome-Level Assembly of the Southern Rock Bream (Oplegnathus fasciatus) Genome Using PacBio and Hi-C Technologies
Source: Front Genet. 2021 Dec 21;12:811798. doi: 10.3389/fgene.2021.811798 (PMC8724560; doi:10.3389/fgene.2021.811798)
Supplement: Supplementary file 3 [file Table7.DOCX]

| **Table S7.** Comparison of gene prediction among *O. fasciatus* and other fish species. | | | | | |
| --- | --- | --- | --- | --- | --- |
| **Species** | **Gene length (bp)** | **Gene number** | **Exon length (bp)** | **Exons number per gene** | **CDS length per gene (bp)** |
| ***Oplegnathus fasciatus*** | 12569.4 | 27015 | 167.47 | 9.27 | 1551.68 |
| ***Gasterosteus aculeatus*** | 9249.29 | 20787 | 160.64 | 10.47 | 1500.17 |
| ***Larimichthys crocea*** | 12818.3 | 23172 | 196.46 | 9.5 | 1456.24 |
| ***Lateolabrax maculatus*** | 13943.8 | 23657 | 254.94 | 10.01 | 1641.5 |
| ***Paralichthys olivaceus*** | 14366.9 | 24665 | 260.51 | 9.74 | 1509.03 |
