# Supplementary material for: Chromosome-Level Assembly of the Southern Rock Bream (Oplegnathus fasciatus) Genome Using PacBio and Hi-C Technologies
Source: Front Genet. 2021 Dec 21;12:811798. doi: 10.3389/fgene.2021.811798 (PMC8724560; doi:10.3389/fgene.2021.811798)
Supplement: Supplementary file 4 [file Table2.DOCX]

| Table S2. Chromosome length summary of *O. fasciatus*. | | |
| --- | --- | --- |
| **Chromosome** | **Chromosome length** | **Scaffold Number** |
| chr1 | 38,983,243 | 3 |
| chr2 | 37,432,500 | 6 |
| chr3 | 36,895,000 | 3 |
| chr4 | 35,461,960 | 7 |
| chr5 | 34,793,000 | 3 |
| chr6 | 34,637,000 | 3 |
| chr7 | 34,545,000 | 1 |
| chr8 | 34,047,500 | 4 |
| chr9 | 33,752,500 | 2 |
| chr10 | 33,001,161 | 3 |
| chr11 | 32,608,429 | 2 |
| chr12 | 31,162,839 | 1 |
| chr13 | 30,900,704 | 2 |
| chr14 | 30,738,500 | 8 |
| chr15 | 30,693,000 | 11 |
| chr16 | 30,573,500 | 6 |
| chr17 | 30,485,500 | 2 |
| chr18 | 29,895,000 | 5 |
| chr19 | 29,696,500 | 4 |
| chr20 | 28,058,000 | 1 |
| chr21 | 28,025,000 | 1 |
| chr22 | 26,675,661 | 4 |
| chr23 | 26,183,234 | 5 |
| chr24 | 19,106,500 | 2 |
| Total | 758,351,231 | 89 |
