# Supplementary material for: Chromosome-Level Assembly of the Southern Rock Bream (Oplegnathus fasciatus) Genome Using PacBio and Hi-C Technologies
Source: Front Genet. 2021 Dec 21;12:811798. doi: 10.3389/fgene.2021.811798 (PMC8724560; doi:10.3389/fgene.2021.811798)
Supplement: Supplementary file 5 [file Table5.DOCX]

| **Table S5.** Comparison of transposable elements in the *O. fasciatus* genome. | | | | | |  |
| --- | --- | --- | --- | --- | --- | --- |
| **Type** | ***Denovo*** | | ***Repbase*** | | ***Total*** | |
|  | **Length** | **% of genome** | **Length** | **% of genome** | **Length** | **% of genome** |
| **DNA** | 114,092,419 | 14.85 | 43,048,948 | 5.6 | 119,055,211 | 15.49 |
| **LINE** | 44,965,005 | 5.85 | 25,745,457 | 3.35 | 46,958,953 | 6.11 |
| **LTR** | 32,908,235 | 4.28 | 11,379,473 | 1.48 | 35,673,168 | 4.64 |
| **SINE** | 1,474,396 | 0.19 | 1,235,899 | 0.16 | 1,440,215 | 0.19 |
| **Total** | 193,440,005 | 25.16 | 81,409,777 | 10.59 | 203,127,547 | 26.43 |
