# Supplementary material for: Chromosome-Level Assembly of the Southern Rock Bream (Oplegnathus fasciatus) Genome Using PacBio and Hi-C Technologies
Source: Front Genet. 2021 Dec 21;12:811798. doi: 10.3389/fgene.2021.811798 (PMC8724560; doi:10.3389/fgene.2021.811798)
Supplement: Supplementary file 6 [file Table3.DOCX]

| **Table S4.** Assessment of genome and annotation completeness by BUSCO. |
| --- |

| Model | Genome | | Proteins | |
| --- | --- | --- | --- | --- |
| Type | Number | Percentage (%) | Number | Percentage (%) |
| Complete BUSCOs (C) | 3560 | 97.8 | 3480 | 95.6 |
| Complete and single-copy BUSCOs (S) | 3531 | 97 | 2418 | 66.4 |
| Complete and duplicated BUSCOs (D) | 29 | 0.8 | 1062 | 29.2 |
| Fragmented BUSCOs (F) | 10 | 0.3 | 57 | 1.6 |
| Missing BUSCOs (M) | 70 | 1.9 | 103 | 2.8 |
| Total BUSCO groups searched | 3640 | 100 | 3640 | 100 |
