# Supplementary material for: Chromosome-Level Assembly of the Southern Rock Bream (Oplegnathus fasciatus) Genome Using PacBio and Hi-C Technologies
Source: Front Genet. 2021 Dec 21;12:811798. doi: 10.3389/fgene.2021.811798 (PMC8724560; doi:10.3389/fgene.2021.811798)
Supplement: Supplementary file 7 [file Table4.DOCX]

| **Table S4.** Prediction of repetitive elements in the *O. fasciatus* genome. | | |
| --- | --- | --- |
| **Type** | **Length (bp)** | **% of genome** |
| **Trf** | 26,298,291 | 3.40 |
| **Repeatmasker** | 235,901,001 | 30.69 |
| **De novo** | 225,024,754 | 29.28 |
