# Supplementary figures and images for: Chromosome-Level Assembly of the Southern Rock Bream (Oplegnathus fasciatus) Genome Using PacBio and Hi-C Technologies
Source: Front Genet. 2021 Dec 21;12:811798. doi: 10.3389/fgene.2021.811798 (PMC8724560; doi:10.3389/fgene.2021.811798)

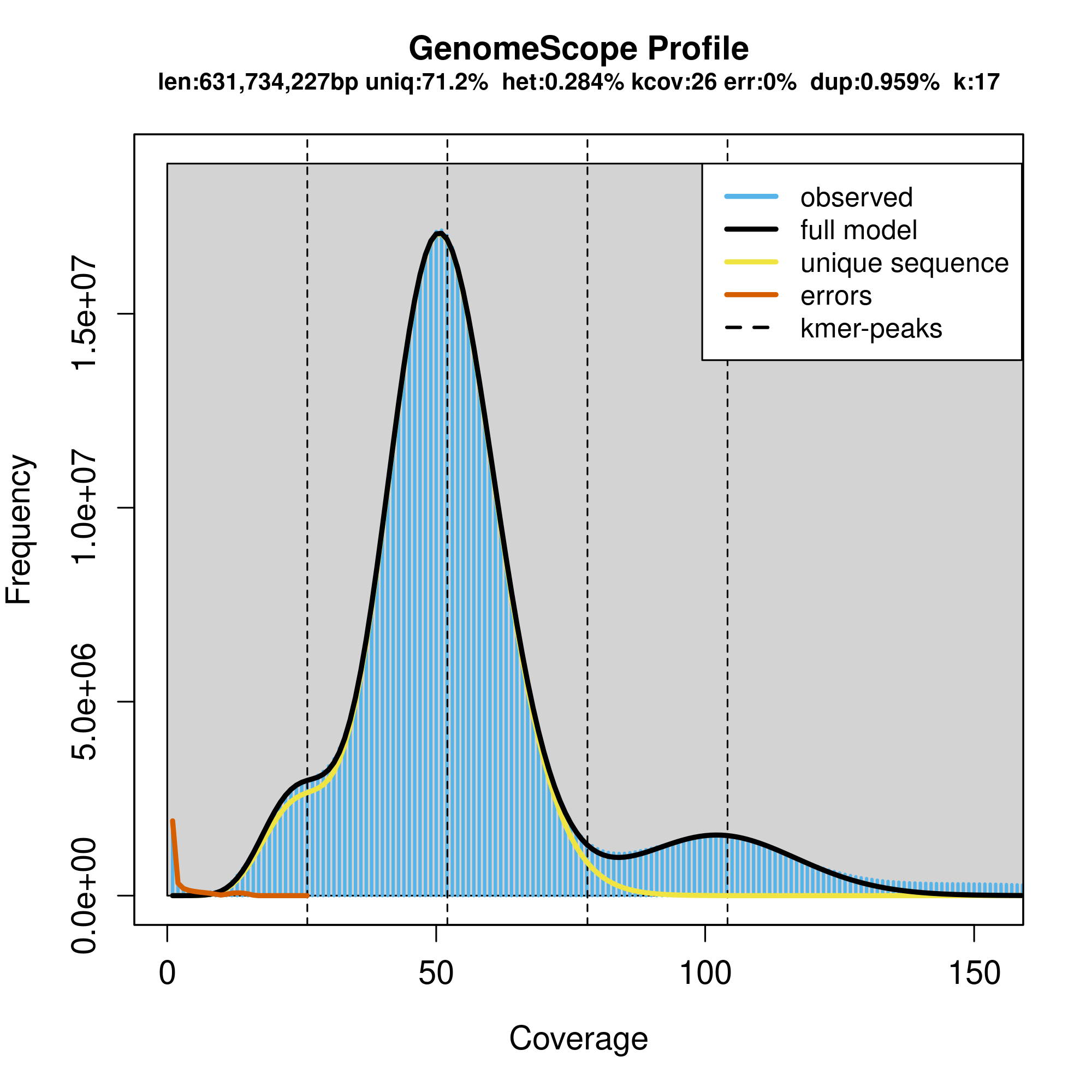

Supplement: Supplementary file 8 [file Image1.PNG]
