# Supplementary material for: Chromosome-Level Assembly of the Southern Rock Bream (Oplegnathus fasciatus) Genome Using PacBio and Hi-C Technologies
Source: Front Genet. 2021 Dec 21;12:811798. doi: 10.3389/fgene.2021.811798 (PMC8724560; doi:10.3389/fgene.2021.811798)
Supplement: Supplementary file 9 [file Table6.DOCX]

| **Table S6.** Functional annotation of the protein-coding genes in *O. fasciatus* genome. | | | | |
| --- | --- | --- | --- | --- |
| **Type** | | **Number** | **Percent (%)** |  |
| **Annotated** | Total | 27,015 | - |  |
|  | NR | 26,739 | 98.98 |  |
|  | TrEMBL | 26,459 | 97.94 |  |
|  | Swissprot | 22,900 | 84.77 |  |
|  | InterPro | 21,228 | 78.58 |  |
|  | GO | 16,144 | 59.76 |  |
|  | KEGG | 16,048 | 59.40 |  |
